# Long-Term Retention Rate of Anakinra in Adult Onset Still’s Disease and Predictive Factors for Treatment Response

**DOI:** 10.3389/fphar.2019.00296

**Published:** 2019-04-02

**Authors:** Antonio Vitale, Giulio Cavalli, Serena Colafrancesco, Roberta Priori, Guido Valesini, Lorenza Maria Argolini, Elena Baldissera, Elena Bartoloni, Daniele Cammelli, Giovanni Canestrari, Jurgen Sota, Elena Cavallaro, Maria Grazia Massaro, Piero Ruscitti, Paola Cipriani, Ginevra De Marchi, Salvatore De Vita, Giacomo Emmi, Gianfranco Ferraccioli, Micol Frassi, Roberto Gerli, Elisa Gremese, Florenzo Iannone, Giovanni Lapadula, Giuseppe Lopalco, Raffaele Manna, Alessandro Mathieu, Carlomaurizio Montecucco, Marta Mosca, Ilaria Piazza, Matteo Piga, Irene Pontikaki, Micol Romano, Silvia Rossi, Maurizio Rossini, Elena Silvestri, Chiara Stagnaro, Rosaria Talarico, Angela Tincani, Ombretta Viapiana, Gianfranco Vitiello, Paola Galozzi, Paolo Sfriso, Carla Gaggiano, Donato Rigante, Lorenzo Dagna, Roberto Giacomelli, Luca Cantarini

**Affiliations:** ^1^Research Centre of Systemic Autoinflammatory Diseases, Behçet’s Disease Clinic and Rheumatology-Ophthalmology Collaborative Uveitis Centre, Department of Medical Sciences, Surgery and Neurosciences, University of Siena, Siena, Italy; ^2^Vita-Salute San Raffaele University, Milan, Italy; ^3^Unit of Immunology, Rheumatology, Allergy and Rare Diseases (UnIRAR), IRCCS San Raffaele Scientific Institute, Milan, Italy; ^4^Rheumatology Unit, Department of Internal Medicine and Medical Specialties, Sapienza University of Rome, Rome, Italy; ^5^Division of Rheumatology, ASST Gaetano Pini, Milan, Italy; ^6^Rheumatology Unit, Department of Medicine, University of Perugia, Perugia, Italy; ^7^Department of Experimental and Clinical Medicine, University of Firenze, Firenze, Italy; ^8^Institute of Rheumatology and Affine Sciences, Division of Rheumatology, Catholic University of the Sacred Heart, Rome, Italy; ^9^Department of Medical and Biological Sciences, Rheumatology Clinic, University of Udine, Udine, Italy; ^10^Periodic Fever Research Center, Institute of Internal Medicine, Catholic University of the Sacred Heart, Fondazione Policlinico A. Gemelli, Rome, Italy; ^11^Department of Biotechnological and Applied Clinical Science, Division of Rheumatology, University of L’Aquila, L’Aquila, Italy; ^12^Rheumatology and Clinical Immunology, Spedali Civili and Department of Clinical and Experimental Sciences, University of Brescia, Brescia, Italy; ^13^Rheumatology Unit, Department of Emergency and Organ Transplantation, University of Bari, Bari, Italy; ^14^Rheumatology Unit, Department of Medical Sciences, University and AOU of Cagliari, Cagliari, Italy; ^15^Department of Rheumatology, IRCCS Policlinico San Matteo Foundation, University of Pavia, Pavia, Italy; ^16^Rheumatology Unit, Department of Clinical and Experimental Medicine, University of Pisa, Pisa, Italy; ^17^Rheumatology Unit, Department of Medicine, University of Verona, Verona, Italy; ^18^Department of Medicine DIMED, Rheumatology Unit, University of Padua, Padua, Italy; ^19^Clinical Pediatrics, Department of Molecular Medicine and Development, University of Siena, Siena, Italy; ^20^Institute of Pediatrics, Università Cattolica Sacro Cuore, Fondazione Policlinico Universitario A. Gemelli I.R.C.C.S., Rome, Italy

**Keywords:** autoinflammatory diseases, systemic onset juvenile idiopathic arthritis, personalized medicine, canakinumab, innovative biotechnologies, interleukin-1

## Abstract

**Background:** Anakinra (ANA) is an effective treatment choice in patients with adult onset Still’s disease (AOSD). Variables affecting treatment survival include loss of efficacy or adverse events, but also the decision to discontinue treatment after long-term clinical remission.

**Objectives:** Aims of this study were: (i) to assess the drug retention rate (DRR) of ANA during a long-term follow-up looking for any difference related to the line of biologic treatment, the concomitant use of conventional disease modifying anti-rheumatic drugs (cDMARDs) and the different type of AOSD (systemic versus chronic articular); (ii) to identify predictive factors of lack of efficacy, loss of efficacy, and ANA withdrawal owing to long-term remission.

**Methods:** AOSD patients classified according with Yamaguchi criteria and treated with ANA were retrospectively enrolled in 18 Italian tertiary Centers. Demographic, laboratory, clinical and therapeutic data related to the start of ANA (*baseline*), the 3-month assessment and the last follow-up visit while on ANA treatment were retrospectively collected and statistically analyzed.

**Results:** One hundred and forty-one AOSD patients (48 males, 93 females) treated with ANA for a mean period of 35.96 ± 36.05 months were enrolled. The overall DRR of ANA was 44.6 and 30.5% at the 60- and 120-month assessments, respectively, with no significant differences between: (i) biologic naïve patients and those previously treated with other biologics (log-rank *p* = 0.97); (ii) monotherapy and concomitant use of cDMARDs (log-rank *p* = 0.45); (iii) systemic and chronic articular types of AOSD (log-rank *p* = 0.67). No variables collected at *baseline* could predict primary inefficacy, while the number of swollen joints at baseline was significantly associated with secondary inefficacy (*p* = 0.01, OR = 1.194, C.I. 1.043–1.367). The typical AOSD skin rash was negatively related with ANA withdrawal owing to long-term remission (*p* = 0.03, OR = 0.224, C.I. 0.058–0.863).

**Conclusion:** Long-term DRR of ANA has been found excellent and is not affected by different lines of biologic treatment, concomitant use of cDMARDs, or type of AOSD. The risk of losing ANA efficacy increases along with the number of swollen joints at the start of therapy, while the typical skin rash is a negative predictor of ANA withdrawal related to sustained remission.

## Introduction

Adult onset Still’s disease (AOSD) is a systemic inflammatory disorder characterized by daily high-spiking fevers, evanescent salmon-colored maculopapular rash, sore throat, serositis, hepatosplenomegaly, lymphadenopathy, myalgia, arthritis, and/or arthralgia. Laboratory investigations usually reveal leukocytosis with neutrophil predominance, increased acute-phase reactants and high levels of serum ferritin, while serum liver enzymes may be often elevated (Pouchot et al., 1991). This condition is frequently considered as the adult counterpart of systemic onset juvenile idiopathic arthritis (SOJIA) (Uppal et al., 1995; Luthi et al., 2002; Martini, 2012).

Clinical presentation of AOSD can be distinguished into two main phenotypes: a “systemic type” characterized by predominantly systemic features including fever, rash, serositis, and organomegaly, and a “chronic articular type” with patients suffering from articular manifestations mimicking rheumatoid arthritis with a polyarticular symmetric pattern. The systemic type can be distinguished into a monocyclic and polycyclic course. A monocyclic AOSD is defined as a single flare lasting from 2 months to 1 year; conversely, the polycyclic course is characterized by recurrent systemic flares with remissions between attacks (Cush et al., 1987).

In the absence of a definitive diagnostic test, diagnosis of AOSD is clinical and requires the exclusion of infectious, neoplastic, autoimmune and other autoinflammatory diseases. Different classification criteria have been proposed for AOSD during the last decades, with the Yamaguchi’s criteria being the most sensitive and the Fautrel’s Criteria the most specific (Yamaguchi et al., 1992; Fautrel et al., 2002). However, Fautrel’s set of criteria includes measurement of glycosylated ferritin, which is not routinely available in many health care facilities.

Disease severity is often determined by using the Pouchot’s score modified by Rau et al. (Pouchot et al., 1991; Rau et al., 2010). This score ranges from 0 to 12 according to the presence or absence of 12 AOSD-related manifestations, each scoring one point.

The pathogenesis of AOSD is already widely unclear with both innate and acquired immunity involved. Nevertheless, based on clinical features and laboratory evidence, this disease has been recently included among polygenic multifactorial autoinflammatory disorders (Hayem, 2009; Rossi-Semerano and Koné-Paut, 2012; Rigante, 2017). In this regard, as for monogenic autoinflammatory diseases, interleukin(IL)-1 blockade has proven to induce a dramatic response in AOSD patients with clinical and laboratory manifestations disappearing within a few days from the start of treatment (Nordström et al., 2008; Giampietro et al., 2013; Cavalli et al., 2015; Ortiz-Sanjuán et al., 2015). On this basis, the IL-1β inhibitor canakinumab has been approved for the treatment of AOSD previously unresponsive to non-steroidal anti-inflammatory drugs (NSAIDs) and corticosteroids. However, most of data on the therapeutic role of IL-1 inhibition in AOSD patients currently relates to the recombinant IL-1 receptor antagonist Anakinra (ANA), associated or not with conventional disease modifying anti-rheumatic drugs (cDMARDs). Sustained and complete efficacy of ANA may also allow a decrease in the frequency of injections until complete withdrawal in some cases (Kong et al., 2010). Nevertheless, beyond the dramatic efficacy reported in most patients, lack and loss of efficacy to ANA have been frequently described (Giampietro et al., 2013; Ortiz-Sanjuán et al., 2015; Rossi-Semerano et al., 2015). Hence, the present study is aimed at assessing the long-term drug retention rate (DRR) of ANA, taking into account the impact of lack and loss of efficacy, adverse events and withdrawal owing to treatment-induced long-term remission. In addition, predictive factors related to lack or loss of efficacy or withdrawal because of long-term remission will be sought among demographic, clinical and laboratory features collected at the start of ANA treatment.

## Materials and Methods

### Patients and Data Collection

Patients enrolled in the present study are almost overlapping with those previously presented in a recent observational study aimed at providing information about efficacy and safety of ANA and Canakinumab when administered in AOSD patients (Colafrancesco et al., 2017).

Demographic, clinical and laboratory data were retrospectively collected from patients suffering from AOSD treated with ANA and attending 18 Italian tertiary Centers. All patients were diagnosed with AOSD according to the Yamaguchi’s criteria (Yamaguchi et al., 1992).

Before starting ANA, patients had undergone a careful laboratory and radiologic screening in order to rule out infections, neoplasms, and other rheumatologic disorders. Patients were closely monitored with weekly clinical and laboratory evaluations during the first month of treatment and then every 3 months or in case of clinical need (disease relapse or safety concerns).

The primary aims of the study were: (i) to assess the long-term DRR of ANA; (ii) to identify any demographic or clinical variable capable to predict the lack or the loss of efficacy to ANA treatment; (iii) to search for predictors of treatment withdrawal owing to sustained remission while on ANA administration. Secondary aims of the study were: (i) to identify any impact on the DRR of ANA by the concomitant use of cDMARDs on the DRR of ANA and by the different biologic line of ANA therapy; (ii) to assess any difference on ANA retention rate according with the different type (systemic versus chronic articular) of AOSD; (iii) to evaluate the long-term cumulative risk for loss of ANA efficacy; (iv) to assess variables related with the treatment duration of ANA; (v) to clarify which AOSD manifestations are more frequently persistent in patients suspending ANA because of lack of efficacy.

The primary endpoints were represented by: (i) the Kaplan–Meier survival estimates obtained during a 120-month follow-up period; (ii) clinical and laboratory variables significantly associated with lack of efficacy, loss of efficacy and ANA withdrawal due to long-term AOSD remission at regression analysis. Secondary endpoints were represented by statistically significant differences in the Kaplan–Meier survival curves between patients treated with ANA as first biologic agent and those previously treated with other biologics; patients concomitantly treated with cDMARDs and those undergoing ANA as monotherapy; patients with systemic AOSD and patients presenting chronic articular disease. Additional secondary endpoints were represented by: the estimates of cumulative probability of loss of ANA efficacy at the reverse Kaplan–Meier analysis; the identification of clinical and laboratory AOSD manifestations significantly more frequent at the 3-month assessment (or at the last ANA administration) in subjects experiencing lack of efficacy compared to the other patients; a statistically significant correlation between treatment duration and the following variables recorded at the start of ANA treatment (*baseline*): age at disease onset, disease duration at the start of ANA, Pouchot score, disease activity score in 28 Joints-C reactive protein (DAS28-CRP), serum ferritin level, and physician’s global assessment.

The demographic, clinical and laboratory variables collected at *baseline* and at the 3-month assessment were: age at AOSD onset, age at diagnosis, disease duration at the start of ANA treatment, number of tender joints, number of swollen joints, DAS28-CRP, previous cDMARDs, previous biologics, concomitant cDMARDs, concomitant corticosteroids, Pouchot score, serum ferritin level, physician’s global assessment of disease activity on a visual analog scale. In addition, the presence or absence of the following clinical and laboratory manifestations were collected: fever, salmon-colored maculopapular skin rash, pleuritis, pneumonia, pericarditis, lymphadenopathy, pharyngodynia, myalgia, arthritis, hepatomegaly, macrophage activation syndrome (MAS), increased liver enzymes levels, leukocytosis, increased erythrocyte sedimentation rate (ESR) and raised CRP. At the last follow-up visit, treatment duration and specific causes leading to ANA withdrawal (primary inefficacy, secondary inefficacy, long-term AOSD remission, safety issues, and loss of compliance) were recorded.

The study protocol was conformed to the tenets of the Declaration of Helsinki and was approved by the local Ethics Committee of the University of Florence (Reference No. 364-16OCT2013). Informed consent was obtained from each patient.

### Definition of Clinical and Laboratory Criteria

The disease was considered “chronic articular” when involvement was prevalently polyarticular with erosive damage and low levels of inflammatory markers. Conversely, AOSD was “systemic” if the patient showed increased inflammatory markers, hyperferritinemia and multi-organ involvement. Disease severity was determined using the modified Pouchot’s score as proposed by Pouchot et al. (1991) and Rau et al. (2010). Systemic AOSD was not distinguished into monocyclic and polycyclic course, as this distinction has been found irrelevant with respect to the prognostic stratification (Cush et al., 1987).

Fever was defined by a temperature higher than 39°C. Diagnosis of pleuritis, pericarditis and pneumonia was based on echographic-radiological documentations; similarly, lymphoadenopathy was confirmed by ultrasound and/or computed tomography (CT) scan in at least two different sites. Hepatomegaly was identified by ultrasound, CT scan or magnetic resonance imaging (MRI). With regard to laboratory evaluations, leukocytosis was defined as a white cell count higher than 15,000/mm^3^ and hyperferritinemia consisted in a serum ferritin level higher than 3,000 ng/ml; ESR and CRP were considered elevated in accordance to each laboratory reference limit.

A *lack of efficacy* (or *primary inefficacy*) was considered as no satisfactory improvement of clinical manifestations during the first weeks of ANA treatment according to physician’s judgment. A *loss of efficacy* (or *secondary inefficacy*) was defined as persistent reappearance of AOSD manifestations leading to ANA withdrawal after a persistent complete response (at least 3 months). Withdrawal of ANA owing to *long-term clinical remission* was based on physician’s judgment; however, a completely asymptomatic period of at least 6 months had been observed in all cases discontinuing ANA due to persistent clinical remission. *Follow-up duration* was defined as the time between the start of ANA and time of ANA withdrawal or the last visit while on ANA treatment.

The study was carried out in accordance with the tenets of the Declaration of Helsinki and the recommendations of the local Ethical Committee (AOUS, Siena, Italy) rules.

### Statistical Analysis

Descriptive statistics included sample size, percentages, means, and standard deviations. After having assessed normality distribution with Shapiro–Wilk test, pair wise comparisons of quantitative data were performed by using unpaired two-tailed *t*-test or Mann–Whitney two tailed *U*-test, as appropriate; Fisher exact test was used for analyzing categorical variables. Correlation analysis was performed using the Spearman test and the Pearson test, as required. Drug survival rates were analyzed by using the Kaplan–Meier plot with “time 0” corresponding to the start of treatment and the “event” being the discontinuation of therapy. Log-rank (Mantel–Cox) test was used to compare different survival curves. The cumulative risk for loss of efficacy was assessed by reverse Kaplan–Meier plot with “time 0” being the start of ANA and the “event” corresponding to treatment withdrawal due to secondary inefficacy.

Binary regression analysis was performed using the backward stepwise to identify baseline clinical and laboratory features associated with lack of efficacy, secondary inefficacy and long-term remission leading to ANA withdrawal as dependent variables. The demographic, clinical and laboratory data collected at *baseline* as listed above were used as independent variables.

Binary regression analysis aimed at identifying variables related to ANA withdrawal due to long-term remission was performed on patients suffering from active AOSD for at least 12 months in order to exclude subjects with a monocyclic disease course.

Odds ratio (OR), its statistical significance and corresponding 95% CI were evaluated for independent variables significantly associated with dependent variables at regression analysis.

The SPSS software, version 24, was used for all statistical computations, always considering a significance level of 95% (*p*-value < 0.05).

## Results

One hundred and forty-one patients (48 males, 93 females) treated with ANA because of AOSD were enrolled in the study. Their demographic and clinical data are summarized in Table 1, while Table 2 provides information about previous and concomitant treatments.

**Table 1:** Demographic, clinical, and laboratory variables referred to the start of Anakinra in the entire cohort of patients enrolled with AOSD.

**Demographic features and disease patterns**	
Age at disease onset, years (mean ± SD)	35.3 ± 17.1
Age at diagnosis, years	37.32 ± 16.95
Disease duration before starting ANA, months	50.4 ± 81.6
Systemic disease pattern	105 (74.5%)
Chronic articular pattern	36 (25.5%)
**Clinical manifestations, *n* (%)**	
Number of tender joints	6.6 ± 6.1
Number of swollen joints	3.0 ± 4.2
DAS28-CRP	4.5 ± 1.5
Pouchot score	5.58 ± 1.92
Body temperature ≥ 39°C	136 (96.5)
Salmon-like skin rash	104 (73.8)
Pleuritis	21 (14.9)
Pericarditis	26 (18.4)
Pneumonia	10 (7.1)
Lymphoadenitis	73 (51.8)
Hepatomegaly	66 (46.8)
Pharingodynia	76 (53.9)
Arthritis	99 (70.2)
MAS during clinical history	12 (8.5)
**Altered laboratory markers, n (%)**	
Increased ESR	120 (85.1)
Increased CRP	129 (91.5)
Leukocytosis	99 (70.2)
Increased serum ferritin	95 (67.4)
Increase liver enzymes	47 (33.3)


*ANA, anakinra; CRP, C-reactive protein; DAS28-CRP, disease activity score in 28 Joints-C-reactive protein; ESR, erythrocyte sedimentation rate; MAS, macrophage activation syndrome; SD, standard deviation*.

**Table 2:** Information about treatment approaches preceding and accompanying treatment with Anakinra (ANA).

	Number of patients (%)
**Previous treatments**	
NSAIDs	97 (68.8)
Corticosteroids	138 (97.9)
cDMARDs	120 (85.1)
Methotrexate	91 (64.5)
Cyclosporine	50 (35.5)
Hydroxychloroquine	30 (21.3)
Colchicine	12 (8.5)
Azathioprine	9 (6.4)
Salazopyrine	8 (5.7)
Leflunomide	5 (3.5)
Gold salts	1 (0.7)
IVIGs	1 (0.7)
Biologic agents	29 (20.6)
Etanercept	20 (14.9)
Infliximab	10 (7.1)
Adalimumab	6 (4.3)
Golimumab	2 (1.4)
Tocilizumab	2 (1.4)
Abatacept	2 (1.4)
Rituximab	2 (1.4)
Certolizumab	1 (0.7)
**Concomitant treatments at baseline**	
cDMARDs	87 (61.7)
Methotrexate	63 (44.7)
Cyclosporine	15 (10.6)
Hydroxychloroquine	12 (8.5)
Colchicine	4 (2.8)
Leflunomide	2 (1.4)
Salazopyrine	2 (1.4)
**Corticosteroids at the last follow-up**	58 (41.1)
**cDMARDs at the last follow-up**	72 (51.1)


*cDMARDs, conventional disease modifying anti-rheumatic drugs; IVIGs, intravenous immunoglobulins; NSAIDs, non-steroidal anti-inflammatory drugs*.

ANA was administered for a mean period of 35.96 ± 36.05 months (median period of 23 months). Withdrawal of ANA was observed in 20 patients (14.2%) because of long-term treatment-induced remission, 16 cases (11.3%) due to primary inefficacy and in 11 (7.8%) cases because of secondary inefficacy. Other 25 (17.7%) patients discontinued ANA because of side effects, as reported in Table 3.

**Table 3:** Adverse events inducing Anakinra withdrawal during the whole follow-up period.

Adverse events	Frequency, n (%)
In-site reactions	10 (7.1)
Generalized urticarial rash	6 (6.3)
Leukopenia/decreased platelet count	2 (1.4)
Angioedema	2 (1.4)
Macrophage activation syndrome	2 (1.4)
Infections	1 (0.7)
Lymphoproliferative disorders	1 (0.7)
Eosinophilia	1 (0.7)


**FIGURE 1:**
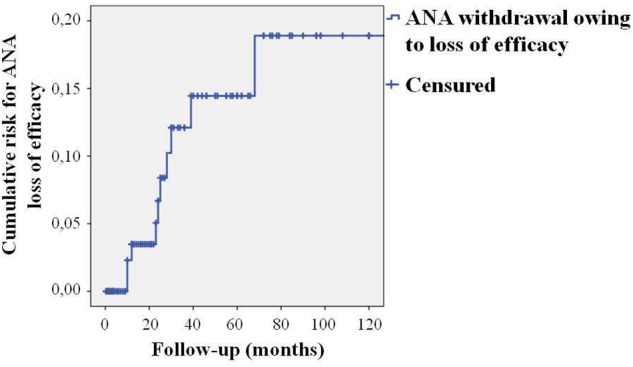
Reverse Kaplan–Meier survival curve showing the cumulative risk for loss of efficacy to Anakinra treatment over time. The “time 0” is represented by the start of ANA treatment; the “event” corresponds to treatment withdrawal due to secondary inefficacy.

Seventeen out of 20 patients suspending ANA due to long-term remission had suffered from active AOSD for at least 12 months. These patients were treated with ANA for a mean period of 35.6 ± 35.4 months. All but two patients experiencing primary inefficacy continued ANA up to the 3-month follow-up visit; secondary inefficacy was observed after a mean period of 35.8 ± 36.1 months of treatment. Figure 1 shows the cumulative risk for loss of efficacy over time, which was 3.4% during the first 12 months, 13.5% at the 60-month assessment, and 17.5% after 120 months.

Regarding dosages employed, 128 (90.8%) patients were administered with standard posology (100 mg/day), 4 (2.8%) patients with higher dosages (200 mg/day), and 9 (6.4%) with lower than standard dosages (100 mg every other day or less).

**FIGURE 2:**
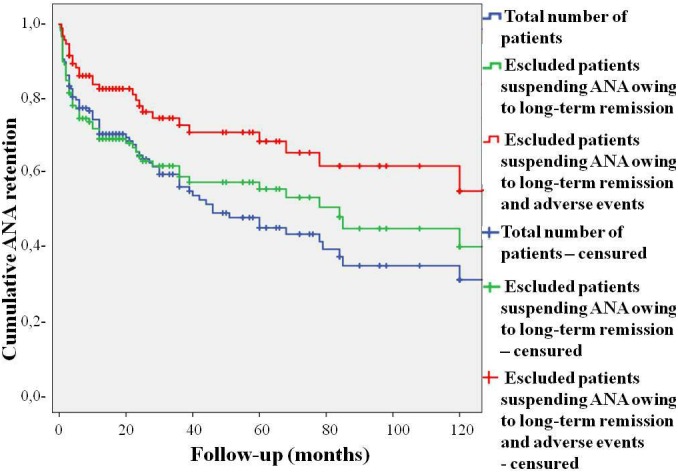
Kaplan–Meier survival curves describing the cumulative retention of Anakinra over the whole follow-up period taking into account all causes of treatment withdrawal (blue line), only adverse events and lack/loss of efficacy (green line), and only lack and loss of efficacy (red line). The “time 0” is represented by the start of ANA treatment; the “event” corresponds to treatment withdrawal.

The overall DRR of ANA was 44.6 and 30.5% at the 60- and 120-month assessments, respectively. After having excluded patients suspending ANA due to long-term remission, the DRR of ANA was 55.2 and 39.5% at the 60- and 120-month assessments, respectively. After excluding also patients discontinuing ANA because of adverse events, the DRR of ANA was 68.2 and 54.6% at the 60- and 120-month evaluations, respectively. These data are illustrated in Figure 2 as Kaplan–Meier survival curves during a 120-month follow-up period.

**FIGURE 3:**
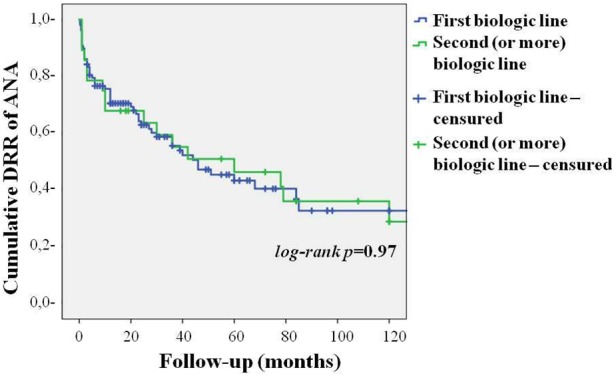
Cumulative DRR of Anakinra in patients with AOSD undergoing their first biologic agent and those previously treated with other biologics.

**FIGURE 4:**
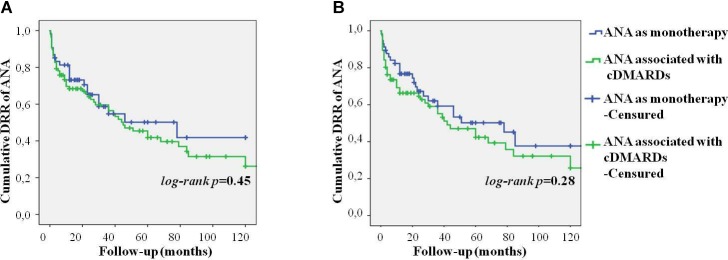
Cumulative DRR of Anakinra in patients with AOSD concomitantly administered with cDMARDs and those treated with IL-1 blockade as monotherapy at the start of ANA treatment **(A)** and at the last follow-up visit **(B)**.

**FIGURE 5:**
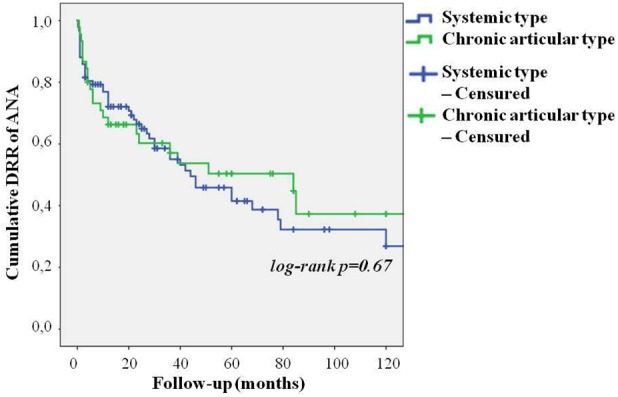
Cumulative DRR of Anakinra in patients with AOSD in both systemic and chronic articular type.

As represented in Figure 3, no differences were found in the DRR of ANA between patients undergoing their first biologic agent and those previously treated with other biologics (log-rank *p* = 0.97). Similarly, no differences were highlighted in the DRR of ANA between patients treated with IL-1 inhibition as monotherapy and those co-administered with cDMARDs both at baseline (log-rank *p* = 0.45) and at the last follow-up (log-rank *p* = 0.28). These results are graphically represented in Figure 4. The lack of statistical significance was maintained when patients suspending ANA due to clinical remission were excluded (log-rank *p* = 0.62 at baseline and log-rank *p* = 0.24 at the last follow-up). No statistically significant differences were identified in the DRR of ANA between the systemic and the chronic articular type (log-rank *p* = 0.67) of AOSD, as also reported in Figure 5.

No significant correlations were identified between treatment duration and age at disease onset (ρ = -0.11, *p* = 0.24), disease duration at the start of ANA (ρ = 0.10, *p* = 0.31), baseline Pouchot score (ρ = 0.03, *p* = 0.77), DAS28-CRP (ρ = 0.06, *p* = 0.56), serum ferritin levels (ρ = 0.09, *p* = 0.34), and physician’s global assessment (ρ = -0.008, *p* = 0.94).

At the binary stepwise regression analysis no variables collected at *baseline* were found to predict primary inefficacy. Conversely, the number of swollen joints at baseline accounted for the only variable capable to predict secondary inefficacy (*p* = 0.01, OR = 1.194, C.I. 1.043–1.367), while neither tender joints nor DAS28-CRP could be included in a predictive model (*p* = 0.30 and *p* = 0.29, respectively).

Binary stepwise regression analysis applied on data collected at *baseline* identified skin rash as the only variable negatively associated with ANA discontinuation due to long-term remission over time (*p* = 0.03, OR = 0.224, C.I. 0.058–0.863).

Among AOSD clinical manifestations, fever (*p* = 0.006), pharyngodynia (*p* = 0.02), tender (*p* = 0.02), and swollen (*p* = 0.021) joints were the clinical manifestations significantly associated with primary inefficacy at the 3-month follow-up visit (or at the last ANA administration). Among laboratory findings, leukocytosis (*p* = 0.004) and increased ESR (*p* = 0.006) observed at the 3-month assessment were significantly associated with primary inefficacy, while CRP (*p* = 0.10) and serum ferritin (*p* = 0.14) did not.

## Discussion

Different clinical and basic research have uncovered the key-role of IL-1 in an extended spectrum of immune dysregulatory conditions, and after showing the dramatic success and safety profile of IL-1 inhibitors in the treatment of cryopyrin-associated periodic syndrome, a complex disease spectrum caused by excessive release of the proinflammatory cytokine IL-1 (Cantarini et al., 2011), many clinicians have been encouraged to a wider use of these drugs in other disorders, including AOSD. During the last decade, ANA has proven to induce a rapid and dramatic improvement of all clinical and laboratory AOSD manifestations, resulting in tapering and discontinuation of concomitant therapy with corticosteroids, NSAIDs, and cDMARDs (Lequerré et al., 2008; Nordström et al., 2008; Giampietro et al., 2013; Ortiz-Sanjuán et al., 2015; Colafrancesco et al., 2017). The efficacy of ANA may also allow a decrease in the frequency of injections until complete withdrawal in some cases (Kong et al., 2010; Colafrancesco et al., 2017). However, lack and loss of efficacy are not rare and require a switch to other treatment approaches, including different IL-1 blockers or IL-6 inhibition (Giampietro et al., 2013; Cavalli et al., 2015; Ortiz-Sanjuán et al., 2015; Rossi-Semerano et al., 2015). Therefore, the present study has been designed to identify any baseline predictor of different outcomes to ANA treatment and evaluate the effectiveness of ANA over time by assessing the long-term DRR with regard to the different causes which might affect survival.

A remarkable DRR was identified during a 10-year study period with 30.5% of patients continuing ANA at the 120-month assessment, when reasons for discontinuation were considered as a whole. This percentage increased to 39.5% when considering only lack/loss of efficacy and adverse events, while more than half of patients continued ANA when the sole lack and loss of efficacy were taken into account. Notably, the risk for loss of efficacy was considerably low with a less than 4% of cumulative risk identified during the first follow-up year. Likewise, the cumulative risk for loss of efficacy increased to only 13.5% during a 5-year period and affected about 1/6 patients during the entire follow-up. As a whole, these results confirm the excellent efficacy of ANA in a higher number of AOSD patients during a substantially long observational period.

In this study the occurrence of adverse events accounts for a leading reason capable to affect the DRR of ANA. However, as also highlighted by Colafrancesco et al. (2017), in our cohort of patients the frequency of adverse events was higher than that reported in previous studies. This could be related to different variables including the longer follow-up period and the relatively low percentage of patients concomitantly treated with cDMARDs. In this regard, Rossi-Semerano et al. (2015) found that background cDMARDs treatment was associated with lower odds of adverse events among patients administered with ANA because of different indications, most of which were represented by AOSD and SOJIA. In addition, the high percentage of MAS cases might be indicative of particular disease severity in our cohort. To the best of our knowledge, this is the first study assessing the DRR of ANA in AOSD patients. However, Woerner et al. (2015) analyzed the DRR of ANA in 51 SOJIA patients undergoing their first biologic agent and in eight cases treated with ANA as second or third biologic option. The estimate of ANA continuation was about 35% at the 100-month assessment in biologic naïve patients when adverse events, ineffectiveness of treatment, loss of response, convenience of use, and patient’s choice were included in the statistical computation. Conversely, the DRR of ANA was strikingly lower among the eight patients previously treated with other biologics, running by 33% at 24 months. A further study proposed by Otten et al. (2013) described a 65% retention of ANA at 12-month follow-up in patients previously exposed to the tumor necrosis factor-α inhibitor etanercept. As inferred from Figure 3, our findings are in line with the results reported by Otten et al. (2013). Similarly, the long-term DRR of ANA obtained from our AOSD patients is very similar to that reported by Woerner et al. (2015) on biologic naïve SOJIA subjects. Nevertheless, the differences identified in the DRR of ANA between patients undergoing their first biologic agent and those previously treated with other biologics are almost unremarkable in our cohort of patients. This discrepancy could be explained by the higher number of patients enrolled in our study.

Noteworthy, the DRR of ANA was not affected by the concomitant use of cDMARDs suggesting that long-term outcome does not differ between monotherapy and combination treatment. However, although other studies have shown similar results (Nigrovic et al., 2011; Giampietro et al., 2013), to date there is no clear evidence that using concomitant cDMARDs does not influence ANA efficacy in SOJIA or AOSD patients and the need to combine ANA with other immunosuppressive drugs should further be explored.

**FIGURE 6:**
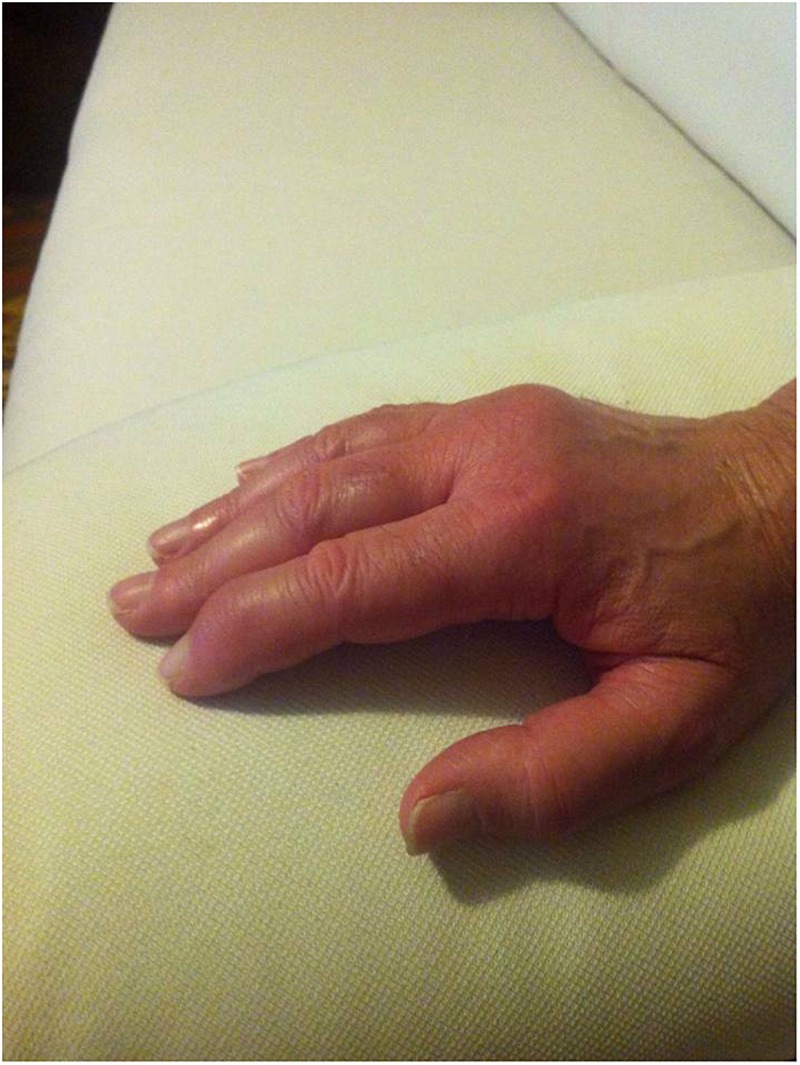
Arthritis involving the right hand in a male patient with AOSD. In particular, the second and third metacarpophalangeal joints, as well as the distal interphalangeal joint of the index finger and the proximal interphalangeal joints of the middle and annular fingers are swollen.

The stepwise binary regression analysis performed on demographic, clinical and laboratory data collected at the start of treatment did not identify any variable capable to anticipate the lack of ANA efficacy; conversely, the number of swollen joints at baseline was the only variable capable to predict secondary inefficacy, while the presence of the typical salmon-like skin rash represented the sole variable associated with the lack of ANA withdrawal owing to sustained clinical remission. Figure 6, 7 show joint involvement and maculopapular skin rash in a patient with AOSD.

**FIGURE 7:**
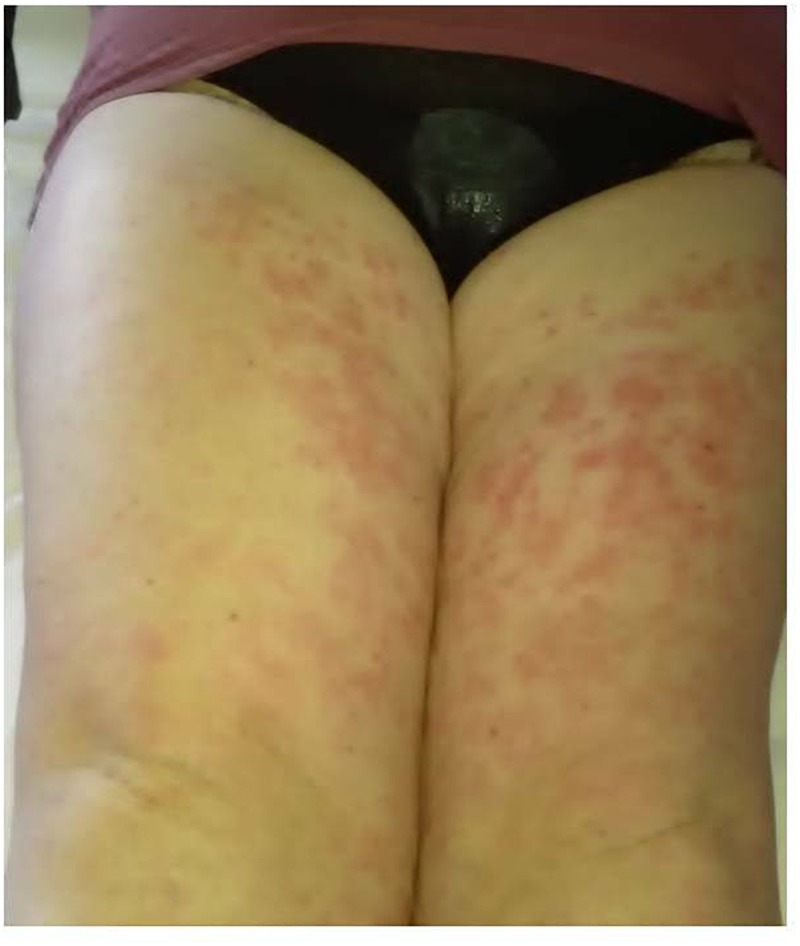
Maculopapular skin rash involving the lower limbs in a female patient suffering from AOSD.

According to published evidences, articular involvement has been frequently described as less responsive than AOSD systemic features (Cavalli et al., 2015; Ortiz-Sanjuán et al., 2015). More in detail, although the frequency of joint symptoms decreases during ANA treatment, articular involvement remains more frequent than other AOSD manifestations (Ortiz-Sanjuán et al., 2015). In addition, articular involvement seems to resolve slowly (within 1 or 2 years), while joint damage could also progress (Lequerré et al., 2008; Lahiri and Teng, 2010; Giampietro et al., 2013). A similar experience has been highlighted also in SOJIA patients: according with Gattorno et al. (2008), subjects with complete response to ANA showed a significantly lower number of active joints compared with patients experiencing incomplete advantage. As a whole, these data seem to corroborate our results on the predictive value of the baseline number of swollen joints on a later loss of efficacy.

Noteworthy, the presence of skin rash at the start of ANA treatment turned out to be a negative predictor of long-term remission leading to ANA withdrawal. This finding could be explained by an even higher IL-1 overproduction in AOSD patients presenting with skin manifestations. In support of this, the persistence of skin lesions has been found to correlate with systemic disease activity, onset of complications, and a more severe prognosis (Fortna et al., 2010; Lee et al., 2012; Yamamoto, 2012; Kikuchi et al., 2014; Sarkar et al., 2014; Cozzi et al., 2016). In this regard, a possible role of the innate immune system of the skin has been suggested in maintaining the degree of systemic inflammation in AOSD (Ruscitti et al., 2016a), while tissue IL-1 levels and other IL-1 family members have proved to be expressed largely in the skin of AOSD patients (Chen et al., 2004; Ruscitti et al., 2016a; Han et al., 2017).

Interestingly, no statistically significant results have been obtained in relationship with AOSD duration before starting ANA treatment. Indeed, according with previous experiences on SOJA patients a shorter time from disease onset to receiving ANA was significantly associated with a favorable response (Nigrovic et al., 2011; Pardeo et al., 2015). On this basis, some authors have speculated about a “window of opportunity” after disease onset during which an early treatment with IL-1 inhibition might have the highest advantage (Nigrovic et al., 2011; Vastert et al., 2014). However, according with our results, disease duration before starting ANA does not predict any outcome and is not correlated with treatment duration in adults with AOSD. Furthermore, no differences were identified in the DRR of ANA on the basis of the different lines of biologic treatment.

Of note, no predictive values were identified neither for the baseline number of tender joints nor for the DAS28-CRP, that is a very useful disease activity score to make an objective, reproducible and comparable evaluation of arthritis activity. Also, no significant differences were identified in the DRR of ANA between AOSD patients suffering from systemic type and those presenting a chronic articular type, while baseline articular involvement did not represent a risk factor for primary inefficacy. On this basis, although the risk for secondary inefficacy increases along with the number of swollen joints at the start of treatment, the presence of arthritis does not affect the short- and long-term response to ANA. Conversely, both systemic and articular features persisted at ANA withdrawal in the subset of patients experiencing lack of efficacy. In particular, fever, pharyngodynia, tender and swollen joints, leukocytosis and increased ESR proved to be the significantly more frequent in the case of ANA failure compared to other patients at the 3-month assessment (or at the last ANA administration).

When we looked for baseline variables capable to correlate with treatment duration, no significant findings were identified among demographic, laboratory or clinimetric data. In particular, neither the Pouchot score, nor the DAS28-CRP nor the physician’s global assessment correlated with the treatment duration. Similarly, no correlations were identified with baseline serum ferritin levels. These findings are of any importance as both the Pouchot score and serum ferritin levels have been correlated with AOSD activity, clinical severity, and prognosis (Efthimiou et al., 2014; Ruscitti et al., 2016b), but do not seem to anticipate the response to ANA treatment.

The main limit of this study is represented by its retrospective nature that prevented to include some interesting quantitative variables for statistical computation. In particular, white cell count, liver enzymes levels, ESR and CRP at baseline were only available as qualitative data (increased/not increased). Consequently, they could not be correlated with treatment duration and were computed as binary information in the stepwise regression analysis. However, all the variables analyzed showed a very low percentage of missing values (<5%) in the data set. In addition, this study is based on a real-life experience and data recorded reflect the everyday management of a wide number of AOSD patients enrolled in 18 different Italian tertiary Centers. This is especially important in reducing recruitment and withdrawal biases, as no defined criteria are currently applicable for starting or stopping ANA in AOSD.

## Conclusion

The present study highlights an excellent long-term DRR of ANA with no significant differences according with the different line of biologic treatment, concomitant use of cDMARDs, or type of AOSD (systemic versus chronic articular). In addition, while no variables have been found to predict primary inefficacy of ANA, the risk for a loss of efficacy increases along with the number of swollen joints at the start of treatment; similarly, the presence of the typical salmon-like maculopapular skin rash at the start of ANA is the only clinical manifestation negatively associated with sustained remission leading to ANA withdrawal over time.

## Data Availability

The datasets generated for this study are available on request to the corresponding author.

## Author Contributions

AV and LC conceived and designed the study and wrote the first draft of the manuscript. AV performed the statistical analysis. All authors have critically reviewed the draft manuscript and approved the submitted version.

## Conflict of Interest Statement

The authors declare that the research was conducted in the absence of any commercial or financial relationships that could be construed as a potential conflict of interest.

## Abbreviations

ANA: anakinra
AOSD: adult onset Still’s disease
cDMARDs: conventional disease modifying anti-rheumatic drugs
CI: confidence interval
CRP: C-reactive protein
CT: computed tomography
DAS28-CRP: disease activity score in 28 Joints-C-reactive protein
DRR: drug retention rate
ESR: eritrocyte sedimentation rate
IL: interleukin
IVIGs: intravenous immunoglobulins
MAS: macrophage activation syndrome
MRI: magnetic resonance imaging
NSAIDs: non-steroidal anti-inflammatory drugs
SOJIA: systemic onset juvenile idiopathic arthritis.
